# A proof of concept for targeting the PrP^C^ - Amyloid β peptide interaction in basal prostate cancer and mesenchymal colon cancer

**DOI:** 10.1038/s41388-022-02430-7

**Published:** 2022-08-12

**Authors:** Sophie Mouillet-Richard, Séverine Martin-Lannerée, Delphine Le Corre, Théo Z. Hirsch, Alexandre Ghazi, Marine Sroussi, Camilla Pilati, Aurélien de Reyniès, Fatima Djouadi, Nicolas Vodovar, Jean-Marie Launay, Pierre Laurent-Puig

**Affiliations:** 1 Centre de Recherche des Cordeliers, Inserm, Sorbonne Université, Université de Paris, F-75006 Paris, France; 2 Laboratoire de Biochimie, Ecole Supérieure de Physique et de Chimie Industrielle de la ville de Paris, Paris, 75005 France; 3 Université Paris Cité and Inserm UMR-S942 MASCOT, Paris, France; 4 Pharma Research Department, F. Hoffmann-La-Roche Ltd., CH-4070 Basel, Switzerland; 5 Institut du Cancer Paris CARPEM, AP-HP, Department of Biology Hôpital Européen Georges Pompidou, F-75015 Paris, France; 6 Present Address: IntegraGen SA Génopole Campus 1, Rue de Henri Desbruères, 91000 Evry, France

**Keywords:** Targeted therapies, Prognostic markers

## Abstract

The cellular prion protein PrP^C^ partners with caveolin-1 (CAV1) in neurodegenerative diseases but whether this interplay occurs in cancer has never been investigated. By leveraging patient and cell line datasets, we uncover a molecular link between PrP^C^ and CAV1 across cancer. Using cell-based assays, we show that PrP^C^ regulates the expression of and interacts with CAV1. PrP^C^ additionally controls the expression of the amyloid precursor protein APP and of the Aβ generating enzyme BACE1, and regulates the levels of Aβ, whose accumulation is a central event in Alzheimer’s disease. We further identify DKK1 and DKK3, involved in both Alzheimer’s disease and cancer progression, as targets of the PrP^C^-dependent axis. Finally, we establish that antibody-mediated blocking of the Aβ-PrP^C^ interaction delays the growth of prostate cancer cell line-derived xenografts and prevents the development of metastases. Our data additionally support an enrichment of the Aβ-PrP^C^-dependent pathway in the basal subtype of prostate cancer, associated with anti-hormonal therapy resistance, and in mesenchymal colon cancer, associated with poor prognosis. Thus, based on a parallel with neurodegenerative diseases, our results bring to light an Aβ-PrP^C^ axis and support the potential of targeting this pathway in patients with selected subtypes of prostate and colon cancer.

## Introduction

Since its discovery in 1985, the cellular prion protein PrP^C^ has been extensively studied for its involvement in a group of fatal neurodegenerative disease known as transmissible spongiform encephalopathies (TSEs) [1]. PrP^C^ is ubiquitously expressed and has since been ascribed a plethora of functions according to the cellular context [2]. Via its GPI anchorage at the outer plasma membrane and its interaction with diverse partners, PrP^C^ can act as a receptor or co-receptor, leading to the mobilization of intracellular signalling cascades [2]. A major PrP^C^ partner in the recruitment of cell signalling pathways is the membrane protein caveolin 1 (CAV1) [2, 3]. In 2009, the discovery that PrP^C^ acts as a receptor for the amyloid Aβ peptide [4], which is produced from the amyloid precursor protein APP following endoproteolytic cleavage and whose accumulation is a central event in Alzheimer’s disease (AD) [5], sparked renewed interest in the signalling function of PrP^C^. It is now well-established that Aβ oligomers hijack PrP^C^-dependent signalling to foster synaptic impairment [2].

A set of studies have allowed to uncover a link between PrP^C^ and cancer [6]. For instance, we recently showed that PrP^C^ is overexpressed in the poor-prognosis subtype of colon cancer and that it controls the expression of a set of genes associated with a mesenchymal phenotype [7]. Along the same line, CAV1 was identified as a specific marker of epithelial to mesenchymal transition (EMT) across cancer cell lines [8]. Altogether, this converging set of data led us to make the provocative assumption that an Aβ-PrP^C^-CAV1 platform may operate in cancer cells and recruit pro-tumorigenic downstream signalling cascades. Here, by combining in silico analysis of several cancer datasets, cell-based assays and mouse xenografts, we delineate an Aβ-PrP^C^-CAV1 axis that sustains cancer growth and metastasis. We further highlight the enrichment of this axis in basal prostate cancer and mesenchymal colon cancer, suggesting its targeting may be beneficial in these specific prostate and colon cancer subtypes.

## Results and discussion

### *PRNP* is highly correlated to *CAV1* across cancer types and PrP^C^ controls caveolin-1 expression in cancer cells

**Fig. 1:**
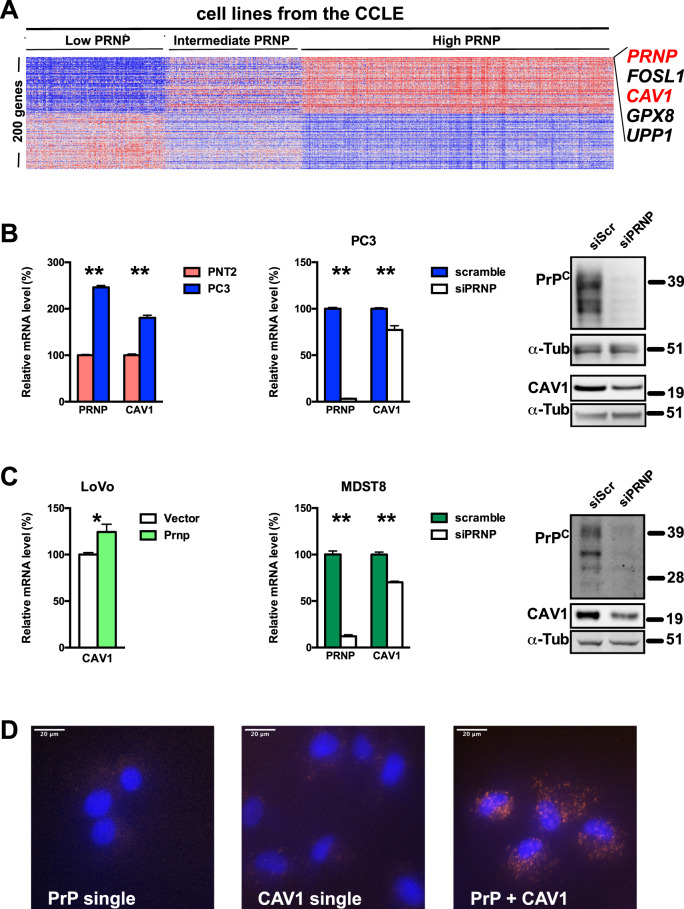
PrP^C^ and Caveolin-1 relationship across cancer. **A** Heatmap showing the top genes most positively and negatively correlated with the expression on *PRNP* in the CCLE. **B** qRT-PCR analysis of the relative expression of *PRNP* and *CAV1* in PC3 prostate cancer cells versus PNT2 normal prostate cells (left panel); relative expression of PrP^C^ and CAV1 mRNA (middle panel) or protein (right panel) in *PRNP*-silenced versus control PC3 prostate cancer cells. **C** qRT-PCR analysis of the relative expression of *CAV1* PrP^C^-overexpressing versus control LoVo colon cancer cells (day 3 post-transfection) (left panel); relative expression of PrP^C^ and CAV1 mRNA (middle panel) or protein (right panel) in *PRNP*-silenced versus control MDST8 colon cancer cells. Results are expressed as means of *n* = 2 independent triplicates of cell preparations ± s.e.m. *p < 0.05, ***p < 0.001 vs. control (Mann-Whitney test). Quantification for western blots is provided in Supplementary Fig. S2, with protein levels normalized to α-tubulin (α-tub). **D** Proximity ligation assay showing co-localization of PrP^C^ and CAV1 in MDST8 cells (orange spots, right panel). Cells incubated with anti-PrP^C^ antibody alone (left panel) or anti-Caveolin-1 antibody alone (middle panel) were used as controls. Nuclei were stained with DAPI.

To probe for a potential link between PrP^C^ and caveolin-1 in cancer, we interrogated published datasets for potential associations between levels of transcripts encoding PrP^C^ (*PRNP*) and caveolin-1 (*CAV1*). We first leveraged the cancer cell line encyclopaedia (CCLE) [9], which provides a unique resource to perform queries on a pan-cancer scale. As shown in Fig. 1A, *CAV1* represented the second most correlated transcript with *PRNP* across the CCLE, suggesting a strong association between the expression of these two genes. We then mined published data sets from The Cancer Genomic Atlas (TCGA) via the Morpheus platform at the Broad Institute (https://software.broadinstitute.org/morpheus/) and found significant correlations between *PRNP* and *CAV1* expression in several cancer types (Supplementary Fig. 1A). The strong correlation between *PRNP* and *CAV-1* was also recovered in two cell line panels (Supplementary Fig. 1A). Of note, the levels of *PRNP* transcripts also strongly correlated with those of CAV1 protein in several of the dataset studied, most notably prostate carcinoma (PRAD) and colon adenocarcinoma (COAD) (Supplementary Fig. 1B).

Next, we turned to cell-based approaches to probe for a functional link between PrP^C^ and CAV1. We measured higher *PRNP* and *CAV1* levels in the PC3 prostate cancer cell line as compared to the PNT2 normal epithelial prostatic cell line (Fig. 1B), and found reduced levels of *CAV1* mRNA and protein when PrP^C^ expression was silenced in PC3 cells (Fig. 1B and Supplementary Fig. 2A). We further exploited the LoVo and MDST8 colon cancer cell lines that express PrP^C^ at low and high levels, respectively [7]. In line with the data obtained in prostate cell lines, overexpression of PrP^C^ in LoVo cells led to an upregulation of *CAV1* mRNA (Fig. 1C) while PrP^C^ knockdown in MDST8 cells caused a reduction in CAV1 mRNA and protein (Fig. 1C and Supplementary Fig. 2B). Finally, PrP^C^ and CAV1 were found to co-localize in MDST8 cells, as evidenced with the Proximity Ligation Assay (Fig. 1D). Altogether, these data highlight regulatory and physical relationships between PrP^C^ and CAV1 in the context of cancer, reminiscent of that described in neuronal cells [2, 3].

### PrP^C^ controls Aβ levels in prostate and colon cancer cells

**Fig. 2:**
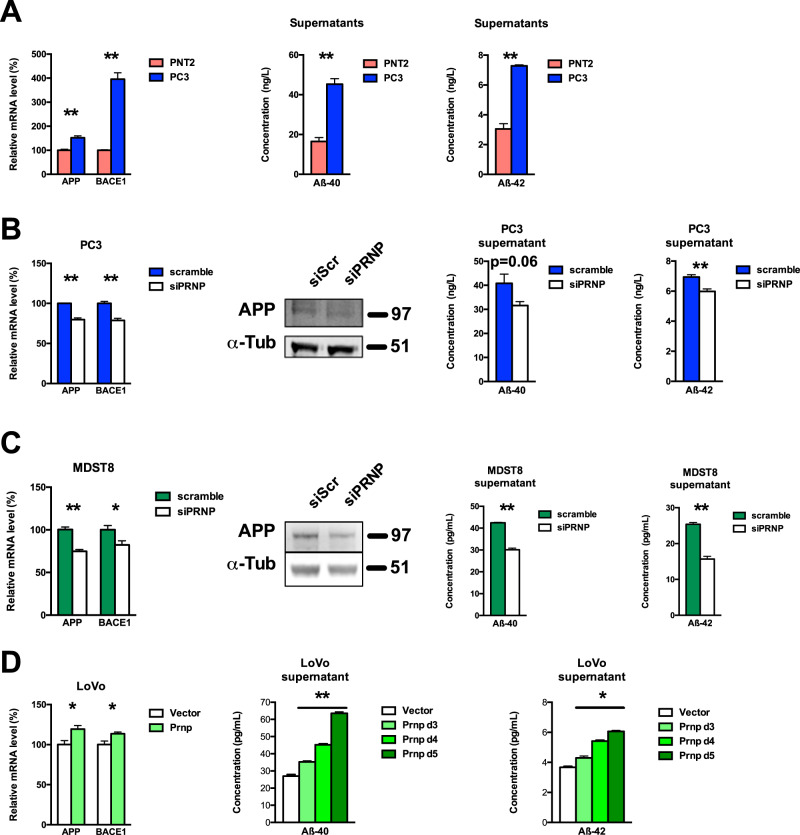
PrP^C^ controls Aβ levels in prostate and colon cancer cells. **A** qRT-PCR analysis of the relative expression of *APP* and *BACE1* in PC3 prostate cancer cells versus PNT2 normal prostate cells (left panel). Extracellular Aβ40 and Aβ42 levels were measured in the supernatants of PNT2 and PC3 cells (right panels). **B** *APP* and *BACE1* mRNA levels, APP protein levels and extracellular Aβ40 and Aβ42 levels were measured in *PRNP*-silenced versus control PC3 prostate cancer cell extracts and supernatants. **C** *APP* and *BACE1* mRNA levels, APP protein levels and extracellular Aβ40 and Aβ42 levels were measured in *PRNP*-silenced versus control MDST8 colon cancer cell extracts and supernatants. **D** *APP* and *BACE1* mRNA levels and extracellular Aβ40 and Aβ42 levels were measured in PrP^C^-overexpressing versus control LoVo colon cancer cell extracts (day 3 post-transfection) and supernatants (days 3, 4 and 5 post-transfection). In **B** and **C**, protein levels were normalized to α-tubulin (α-tub). Results are expressed as means of *n* = 2 independent triplicates of cell preparations ± s.e.m. *p < 0.05, **p < 0.01 vs. control (Mann-Whitney test except for D middle and right panels Kruskal-Wallis and posthoc Wilcoxon rank-sum test with Holm’s correction). Quantification for western blots is provided in Supplementary Fig. S2C, D.

Since *PRNP* mRNA and CAV1 mRNA and protein levels consistently correlated with those of transcripts encoding *APP* in the CCLE (Supplementary Fig. 1C), we next examined a potential link between PrP^C^ and APP and its proteolytic fragments in prostate and colon cancer cells. As shown in Fig. 2A, PC3 cells expressed higher levels of mRNAs encoding *APP* as well as *BACE1*, a protease involved in the processing of APP and the generation of Aβ peptides [10], when compared to PNT2 cells. Accordingly, we measured higher levels of the Aβ-40 and Aβ-42 peptides in the supernatants of PC3 cells, when compared to PNT2 cells (Fig. 2A). Silencing of PrP^C^ (48 h) in the PC3 cell line was associated with a reduction in APP mRNA and protein, *BACE1* mRNA, as well as extracellular Aβ-40 and Aβ-42 concentrations (Fig. 2B and Supplementary Fig. 2C). As observed with PC3 cells, APP mRNA and protein, *BACE1* mRNA, and extracellular Aβ-40 and Aβ-42 concentrations were all reduced in MDST8 cells after PrP^C^ knockdown (Fig. 2C and Supplementary Fig. 2D). Finally, in the LoVo colon cell line, PrP^C^ overexpression promoted an upregulation of *APP* and *BACE1* transcripts (Fig. 2D). Of note, we found that the concentrations of Aβ-40 and Aβ-42 peptides in the supernatants of LoVo cells progressively increased along a 3 to 5-day window post-transfection with the PrP^C^-encoding plasmid (Fig. 2D and Supplementary Fig. 2E). Altogether, these data indicate that PrP^C^ controls the expression of APP, as well as that of BACE1, the main sheddase responsible for the production of Aβ peptides [10]. These observations differ with several studies reporting a negative correlation between PrP^C^ and BACE1 or Aβ levels [11–14]. Yet, they are in agreement with the observed positive correlation between PrP^C^ and APP levels in the brain of transgenic mice [15]. They also fully fit in with the reported promotion of APP cleavage by PrP^C^ in the brain through the upregulation of BACE1 expression [16], as well as the recently described PrP^C^-dependent secretion of Aβ in cell lines [17]. Accordingly, our gain and loss of function experiments strongly support a control of PrP^C^ over the soluble concentrations of Aβ peptides in cancer cells.

### Targeting the Aβ-PrP^C^ interaction in vitro reduces the expression of a set of genes associated with mesenchymal features of cancer cells and lowers the levels of extracellular Aβ40, Aβ42 and TGFβ1

**Fig. 3:**
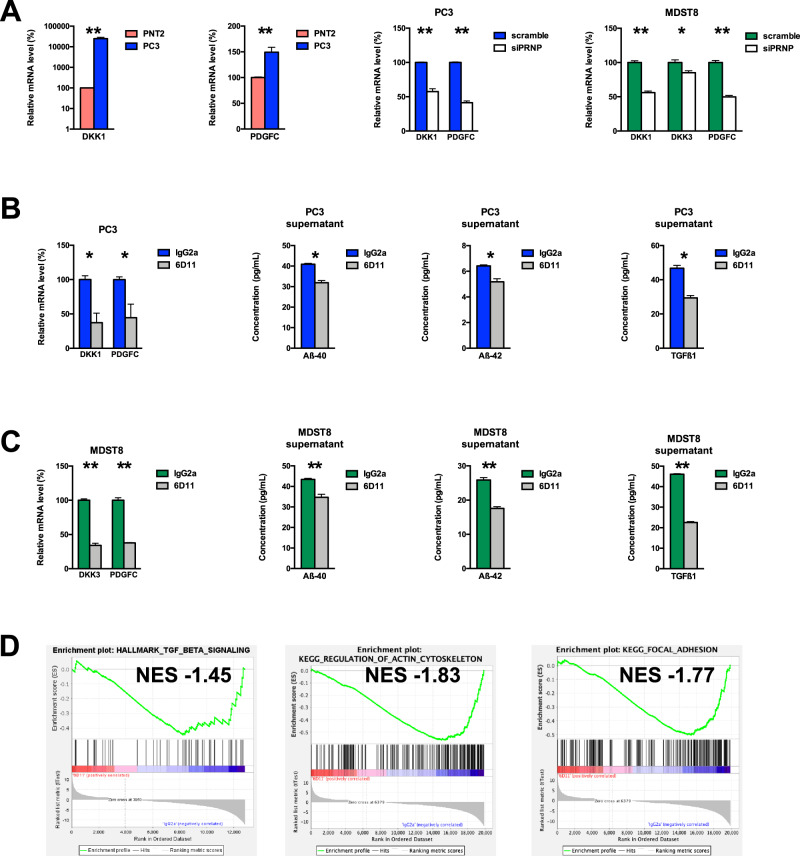
Identification of potential Aβ-PrP^C^ targets and impact of antibody-mediated blockade of the Aβ-PrP^C^ interaction. **A** qRT-PCR analysis of the relative expression of *DKK1* and *PDGFC* in PC3 prostate cancer cells versus PNT2 normal prostate cells (left panels) as well as in *PRNP*-silenced versus control PC3 cells (middle panel) and qRT-PCR analysis of the relative expression of *DKK1, DKK3* and *PDGFC* in *PRNP*-silenced versus control MDST8 cells (right panel). **B** *DKK1* and *PDGFC* mRNA levels and extracellular Aβ40, Aβ42 and TGFβ levels were measured in cell extracts and supernatants of PC3 prostate cancer cells exposed to 6D11 antibodies versus control isotype antibodies. **C** *DKK3* and *PDGFC* mRNA levels and extracellular Aβ40, Aβ42 and TGFβ levels were measured in cell extracts and supernatants of MDST8 colon cancer cells exposed to 6D11 antibodies versus control isotype antibodies. Results are expressed as means of *n* = 2 independent triplicates of cell preparations (except for **A** and **B** *n* = 2 independent duplicates of cell preparations) ± s.e.m. *p < 0.05, ***p < 0.001 vs. control (Mann-Whitney test). **D** GSEA analysis showing the downregulation of the TGFβ signalling, regulation of actin cytoskeleton and focal adhesion signatures in 6D11-treated versus control MDST8 cells. NES normalized enrichment score.

Next, we searched for candidate targets downstream of the PrP^C^-CAV1 module that may potentially be recruited upon binding of Aβ to PrP^C^. Among the genes whose levels significantly correlated with those of *PRNP* mRNA, *APP* mRNA and *CAV1* mRNA and protein in the CCLE, we selected *DKK1*, *DKK3* and *PDGFC* for further analysis (Supplementary Fig. 3), in view of the potential link between DKK1/DKK3 and Alzheimer’s disease [18, 19], their contribution to tumour progression by activating the tumour microenvironment [20, 21] and the PrP^C^-dependent regulation of *PDGFC* in colon cancer cells [7]. We measured increased levels of *DKK1* and *PDGFC* mRNA in PC3 versus PNT2 cells, which were strongly reduced after PrP^C^ knockdown in PC3 cells (Fig. 3A). In line with this, PrP^C^-silenced MDST8 cells exhibited reduced mRNA levels of *DKK1*, *DKK3* and *PDGFC* (Fig. 3A).

Next, we probed whether blockade of the Aβ-PrP^C^ interaction would impact on *DKK1/3* and *PDGFC* transcript levels. To this end, cells were exposed to the 6D11 antibody (10 µg/mL, 72 h) targeting the 92-110 epitope within PrP^C^ that binds Aβ [4]. Treatment of PC3 cells with 6D11 induced a significant reduction in *DKK1* and *PDGFC* transcript levels (Fig. 3B). We further observed a decrease in the levels Aβ-40 and Aβ-42 in the supernatants of PC3 cells treated with 6D11, as well as that of TGFβ1, which we previously showed to be controlled by PrP^C^ in colon cancer cells [7] (Fig. 3B). In line with the above data, *DKK3* and *PDGFC* transcripts were reduced in MDST8 cells after treatment with 6D11 (Fig. 3C). We further explored some PrP^C^-regulated genes described above (*BACE1*, *CAV1*) or that we previously showed to be associated with the mesenchymal phenotype of MDST8 cells (*IDO1*, *TGFB1*, *ZEB1*) [7, 22], and found that expression of this set of genes was robustly decreased upon blockade of the PrP^C^- Aβ interaction with 6D11 antibodies (Supplementary Fig. 4A). As with PC3 cells, the levels of Aβ-40, Aβ-42 and TGFβ1 were decreased in the supernatants of MDST8 treated with 6D11 (Fig. 3C). In contrast to the effects observed with 6D11, treatment of PC3 cells with the Sha31 antibody that does not interfere with the Aβ-PrP^C^ interaction induced only a mild decrease in *DKK1* mRNA and was neutral to *PDGFC* mRNA (Supplementary Fig. S4B). As for MDST8 cells, exposure to the Sha31 antibody promoted an increase in *DKK3* and *PDGFC* levels (Supplementary Fig. 4C), in agreement with Sha31 mimicking a ligand-induced activation of PrP^C^ [2]. A more global profiling of the transcriptome of MDST8 cells treated with 6D11 through RNAseq followed by GSEA analysis revealed that 3 gene signatures, TGFβ signalling, regulation of actin cytoskeleton and focal adhesion, which are significantly correlated with *PRNP* gene expression in colon cancer [7] and data not shown), were downregulated in response to 6D11 treatment (Fig. 3D). Whether 6D11 promotes the internalization of PrP^C^, as described by Pankiewicz et al. [23] or its shedding, as depicted by Linsenmeier et al. [24], or operates through another mode of action warrants further investigation. As a whole, our data suggest that Aβ-PrP^C^ signalling controls the expression of DKK1/3 and PDGFC as well as Aβ and TGFβ levels in prostate and colon cancer cells and regulates pathways involved in the mesenchymal subtype of colon cancer.

### Blockade of the Aβ-PrP^C^ interaction reduces the growth of prostate cancer cells in vivo and the Aβ-PrP^C^ connection has translational relevance in prostate and colon cancer patients

**Fig. 4:**
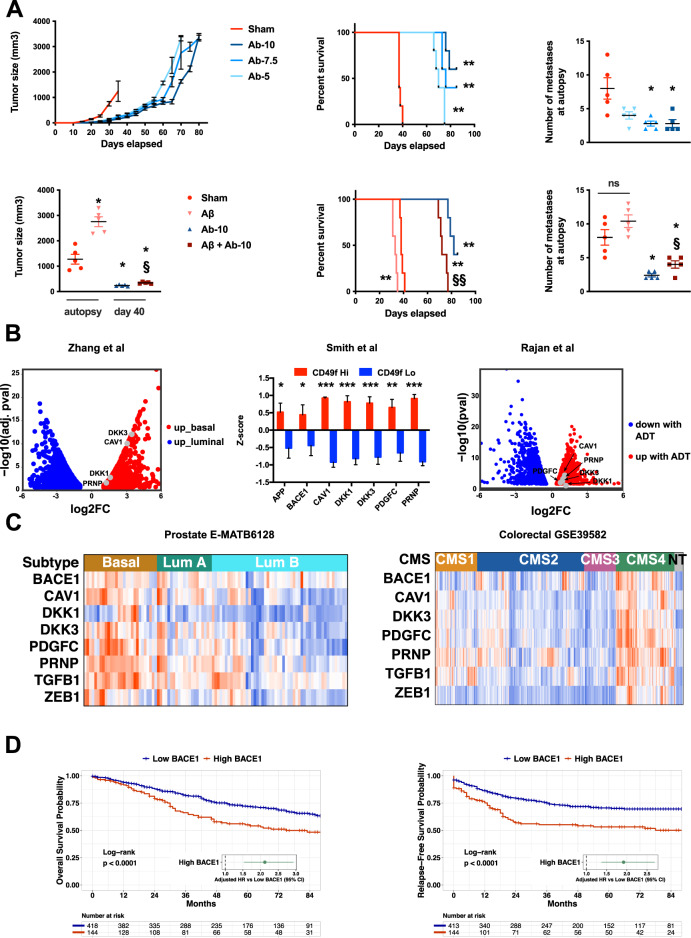
The Aβ-PrP^C^ axis can be targeted in vivo and has clinical relevance in basal prostate cancer and mesenchymal colon cancer. **A** Top panels. Tumour growth, survival curves and number of metastases in mice bearing PC3 xenografts and treated with 5, 7.5 or 10 mg per kg of 6D11 antibody (Ab-5, Ab-7.5, Ab-10, respectively) versus control mice (Sham). Bottom panels. Tumour size (measured at autopsy for the sham and Aβ groups, and at day 40 for the Ab-10 and the Aβ + Ab-10 groups), survival curves and number of metastases in mice bearing PC3 xenografts and treated with recombinant Aβ, 6D11 antibody (at 10 mg per kg, Ab-10) or both Aβ and 6D11 antibody (Aβ + Ab-10) versus control mice (Sham). Data for tumour size and number of metastases are expressed as means ± s.e.m. of *n* = 5 values (*p < 0.05 and **p < 0.01 versus Sham, ^§^*p* < 0.05 and ^§§^*p* < 0.01 versus Aβ alone, Kruskal-Wallis and posthoc Wilcoxon rank-sum test with Holm’s correction for tumour size and number of metastases, log-rank test for survival curves. ns not significant). **B** Volcano plot showing the enrichment of *CAV1*, *DKK1*, *DKK3* and *PRNP* transcripts in basal versus luminal benign prostate cancers from the Zhang study [26] (left panel); Enrichment of *APP*, *BACE1*, *CAV1*, *DKK1*, *DKK3*, *PDGFC* and *PRNP* transcripts in CD49f^Hi^ versus CD49f^Lo^ prostate tissue from the Smith study [27] (middle panel) (*p < 0.05 and ***p < 0.001 versus CD49f^Lo^, two-tailed *t*-test); Volcano plot showing the upregulation of *CAV1*, *DKK1*, *DKK3*, *PDGFC* and *PRNP* transcripts after ADT in the Rajan study [32] (right panel). **C** Heatmaps showing the distribution of *PRNP*-associated genes in prostate cancer patients (E-MATB6128 data set) according to the PAM50 classification by Zhao [29] (left panel) or in colon cancer patients (GSE39582 dataset) according to the CMS classification by Guinney [31] (right panel). LumA: Luminal A. Lum B: Luminal B. NT: non tumour. See Supplementary Fig. S5 and S7 for statistics. **D** Kaplan-Meier overall survival (OS) (left panel) and relapse free survival (RFS) (right panel) according to high and low *BACE1* gene expression was determined in colon cancer patients of the GSE39582 dataset. Hazard ratios were adjusted for TNM stage, MMR status and adjuvant chemotherapy.

The above results prompted us to target the Aβ-PrP^C^ interaction in an in vivo model of tumorigenesis. To this end, we studied the effect of 6D11 on the growth of PC3 xenografts in mice (see Materials and Methods). Castrated mice bearing palpable tumours were treated twice weekly with PBS or 6D11 at various doses (5, 7.5, or 10 mg per kg of body weight). As shown in Fig. 4A and Supplementary Fig. 5A, B, the 6D11 antibody markedly reduced tumour growth at all doses, when compared to controls. This effect was prolonged beyond 60 days at the highest dose. While the growth of tumours strongly resumed around day 60 depending on the dose received, prolonged administration of 6D11 promoted a > 100% improvement in the mean survival time of PC3-grafted mice (Fig. 4A). Importantly, the metastatic burden in xenografted mice was strongly reduced upon 6D11 treatment (Fig. 4A and Supplementary Fig. 5C). Furthermore, we found that treating PC3-grafted mice with recombinant Aβ boosted tumour growth and shortened survival (Fig. 4A). Remarkably, these effects were counteracted upon concomitant treatment with 6D11 antibodies, which also drastically reduced metastatic burden (Fig. 4A). Thus, these data establish a tumour-promoting effect of Aβ, which has to be brought together with the recent identification of melanoma-secreted Aβ as a promoter of brain metastasis [25]. They also demonstrate that abrogating the Aβ-PrP^C^ interaction is beneficial in a pre-clinical prostate cancer model. Next, we interrogated public datasets to evaluate the potential translational relevance of the PrP-dependent axis in prostate cancer. Interestingly, we found an enrichment of *PRNP*, *CAV1*, as well as *DKK1* and *DKK3* mRNA levels in basal versus luminal benign prostate biopsies in the Zhang dataset [26] (Fig. 4B). Of note, the basal cell signature was shown to be associated with aggressive prostate cancer [26]. Along the same line, *PRNP*, *CAV1*, *DKK1*, *DKK3* and *PDGFC* were all significantly enriched in the basal cell population from benign and cancer prostate tissue, characterized by high expression of the cell surface marker CD49f [27] (Fig. 4B). As in [26], Smith et al. argued that the CD49f^Hi^-associated gene signature is a hallmark of aggressive prostate cancer [27]. Furthermore, analysis of matched samples before and after androgen-deprivation therapy (ADT) revealed an increase in the same set of genes after ADT [28] (Fig. 4B). Finally, we sought to assess the distribution of the various genes of interest according to the recently described molecular classification of prostate cancer samples into Luminal A, Luminal B or Basal subtypes [29]. Applying the PAM50 classifier designed by [29] to the dataset of Kamoun et al. [30], we observed an enrichment of the various genes of the pathway in tumours of the basal subtype compared to those of the luminal B subtype (Fig. 4C). *PRNP*, *PDGFC* and *TGFB1* mRNAs were also significantly higher in basal versus luminal A tumours (Fig. 4C and Supplementary Fig. 6). The enrichment of the PrP^C^-dependent axis in the basal versus luminal B subtype was also recovered in TCGA prostate cancer samples (Supplementary Fig. 7). Of note, although patients with basal and luminal A prostate cancer have a better prognosis than those with luminal B prostate cancer, they were shown to respond poorly to ADT treatment [29]. As for colon cancer, we had previously analysed the expression of *PRNP* according to the consensus molecular classification defined by Guinney et al. [31] and we had documented an enrichment of *PRNP* transcripts in the poor-prognosis mesenchymal subtype referred to as CMS4 [7], associated with poor prognosis [31]. Here, in line with the above findings, we monitored a significant enrichment of the PrP^C^-dependent network in tumours of the CMS4 subtype (Fig. 4C and Supplementary Fig. 8). Finally, the expression of the *BACE1* gene was highly prognostic for overall survival and relapse-free survival in the GSE39582 cohort both as a continuous variable in univariate (Hazard Ratio HR = 1.85, 95% CI = 1.29 to 2.66, *p* < 0.001 for OS and HR = 2.39, 95% CI = 1.65–3.46, *p* < 0.001 for RFS) and multivariate (HR = 1.88, 95% CI = 1.27–2.78, *p* < 0.01 for OS and HR = 1.99, 95% CI = 1.34–2.95, *p* < 0.001 for RFS) analysis and categorical variable (dataset GSE39582) (Fig. 4D), as well as in a large validation cohort (Supplementary Fig. 9). In summary, the Aβ-PrP^C^-dependent axis appears to represent a hallmark of basal prostate cancer and mesenchymal colon cancer, thus opening new avenues for the development of therapeutic strategies specifically targeting these cancer molecular subgroups.

Taken together, our study brings compelling evidence supporting the activation of an Aβ-PrP^C^-CAV1 dependent signalling pathway in specific subtypes of prostate and colon cancer. It provides a proof of concept that targeting the Aβ-PrP^C^ interaction has beneficial effect and warrants testing the impact of Aβ-PrP^C^ binding blockade in combination with standard chemotherapy.

## Materials and Methods

See Supplementary Information for further details.

## Supplementary information

supplementary material

supplementary Figure 1

supplementary Figure 2

supplementary Figure 3

supplementary Figure 4

supplementary Figure 5

supplementary Figure 6

supplementary Figure 7

supplementary Figure 8

supplementary Figure 9
