# Supplementary material for: A proof of concept for targeting the PrPC - Amyloid β peptide interaction in basal prostate cancer and mesenchymal colon cancer
Source: Oncogene. 2022 Aug 12;41(38):4397–404. doi: 10.1038/s41388-022-02430-7 (PMC9481457; doi:10.1038/s41388-022-02430-7)
Supplement: Supplementary file 1 — supplementary material [file 41388_2022_2430_MOESM1_ESM.docx]

**Supplementary materials to:**

**A proof of concept for targeting the PrP^C^ - Amyloid ß peptide interaction in basal prostate cancer and mesenchymal colon cancer**

by Sophie Mouillet-Richard et al.

Supplementary Materials and Methods

Legends to Supplementary Figures 1 to 9

Supplementary Table 1

Supplementary references

**Supplementary Materials and Methods**

**Reagents**

All tissue culture reagents were from Invitrogen (Carlsbad, CA, USA). Mouse monoclonal antibodies against α-tubulin (T9026) and human nuclei (MAB1281) were from Sigma-Aldrich (St. Louis, MO, USA). Mouse monoclonal antibody against APP (22C11) was from Invitrogen. Mouse monoclonal antibodies Sha31 and 12F10 against PrP^C^ were from SPI-Bio (Montigny Le Bretonneux, France). Mouse monoclonal antibody 6D11 against PrP^C^ and IgG isotype control antibodies were from Biolegend (San Diego, CA, USA). Rabbit polyclonal antibody against CAV1 (#3267) was from Cell Signaling (Danvers, MA, USA). Mouse monoclonal antibody against YAP/TAZ was from Santa Cruz (sc-101199). Aβ42 trimers were synthesized and purified in the Roche AG Chemistry Department by size-exclusion chromatography, reversed-phase high performance liquid chromatography and cross linking [1].

**Cell culture and treatment**

The human prostate PNT2 and PC3 cell lines and the colon MDST8 and LoVo cell lines were purchased from Sigma, which provided cell authentication. PNT2 and PC3 cells were grown in RPMI-1640 medium supplemented with 2mM glutamine and 10% fetal bovine serum. MDST8 cells were grown in DMEM with 10% fetal bovine serum. LoVo cells were grown in F-12K medium supplemented with 10% fetal bovine serum. All cell lines were grown at 37 °C and 5% CO_2_ in a humidified incubator and regularly tested for mycoplasma contamination. For transient siRNA-mediated silencing, cells were transfected with siRNA sequences (30 nM) as in [2]. For PrP^C^ overexpression, LoVo cells were transfected with 2.5 µg of the pcDNA3-prnp plasmid that expresses mouse *Prnp* (kind gift of Pr. Sylvain Lehmann) using the Lipofectamine 3000 reagent according to the manufacturer’s instructions (Invitrogen) as in [2]. The corresponding empty vector was used as control.

For antibody exposure, cells were washed twice in PBS and incubated in culture medium containing 1% serum with 10 µg/ml antibody for 72h. Cells treated with control IgG isotype antibodies were used as reference. We checked that treatment with control isotype antibodies had no impact on the various readouts, as compared with cells incubated in medium with 1% serum without antibody.

### Preparation of cell supernatants, protein extracts and western blot analyses

### The cells supernatants were collected, centrifuged for 5min at 1,500 g and immediately snap frozen at -80°C. Cells were washed in PBS and incubated for 30 minutes at 4°C in NaDOC lysis buffer [50 mM Tris·HCl (pH 7.4)/150 mM NaCl/5 mM EDTA/0.5% Triton X-100/0.5% sodium deoxycholate], a mixture of phosphatase (Thermo-Scientific, Waltham, MA, USA) and protease (Roche, Mannheim, Germany) inhibitors. Extracts were centrifuged at 14,000 x *g* for 15 minutes. Protein concentrations in the supernatant were measured by using the bicinchoninic acid method (Pierce, Rockford, IL, USA). Protein extracts (15 µg) were separated by 4-12% SDS-PAGE (Invitrogen) and transferred to nitrocellulose membranes (iBlot, Invitrogen). Membranes were blocked with SEABLOCK blocking buffer (Thermo-Scientific) for 1 hour at room temperature and then incubated overnight at 4°C with the primary antibody. Bound antibody was revealed by infrared detection using a secondary antibody coupled to IRDye fluorophores (Li-Cor biosciences, Lincoln, NE, USA). Quantification was performed with the Odyssey Infrared Imaging System (Li-Cor biosciences).

**ELISA**

ELISA for soluble TGFβ1 was from Biolegend (San Diego, CA, USA).

A-β40 and A-β42 were quantified in cell supernatants through ELISA (My Biosource MBS724063 and MBS846485).

**Isolation of total RNA and RT-PCR analysis**

Total RNA was isolated by using the RNeasy extraction kit (Qiagen, Limburg, Netherlands), as per manufacturer's instructions. For reverse transcriptase-polymerase chain reaction (RT-PCR) analysis, first-strand cDNA was synthesized with oligo(dT) primer and random hexamers, using the High-capacity cDNA Reverse Transcription (Applied Biosystems) according to the manufacturer's protocol. Real-time PCR was performed using Absolute QPCR SYBR Green ROX Mix (Thermo-Scientific, Waltham, MA, USA) on a ABI PRISM 7900HT (Applied Biosystems, Life Technologies Corporation, Carlsbad, CA, USA). Real-time PCR analyses were performed with the SDS software 2.3 (Applied Biosystems). Primers used for the PCR reactions are shown on Supplementary Table S1. Results are expressed as a relative quantification of a target gene transcript normalized to the *RPL13A* housekeeping gene using the ∆∆Ct method.

**RNAseq analysis**

Libraries were prepared from 200 ng of total RNA using the QuantSeq 3’ mRNA-Seq Library Prep Kit FWD for Illumina from Lexogen, and sequenced on a Novaseq 6000 instrument. Bioinformatic analyses were performed with R 4.0.4. RNAseq data were processed with Kallisto program [3] (version 0.46.1) to quantify abundances of transcripts. The genome index was previously built on Ensembl Transcriptomes v96. The Kallisto output files were read and analysed with Sleuth package [4] (version 0.30.0). Once converted to count matrix, data were normalized, log transformed and scaled. The GSEA [5] was performed using the Broad Institute platform (http://www.broadinstitute.org/gsea/index.jsp; Version 2.0.14).

**Proximity ligation assay**

MDST8 seeded on Labteck chambers (Nunc, Rochester, NY, USA) were fixed with 4% paraformaldehyde in PBS and permeabilized with 0.1% Triton in PBS containing 20 mM glycine for 15 min. Proximity ligation assay was performed using the Duolink in Situ Orange Starter Kit Mouse/Rabbit from Sigma, according to manufacturer’s instructions. Primary antibodies were used at 1/20 (12F10) and 1/200 (CAV1) dilution and were incubated for 1 hour at room temperature. Images were recorded using a Zeiss Axio Observer Z1 at X40 magnification.

**In vivo studies**

All in vivo experiments were performed according to approved protocols from the Committee on the Ethics of Animal Experiments from Basel University. PC3-luciferase cells (1x10^6^ cells) in 50% Cultrex (Trevigen) were injected subcutaneously into 6-8-week-old castrated SCID male mice. Starting from day 10 post-implantation, mice (n=5 for each group, randomly assigned) received either PBS or 6D11 anti-PrP antibody (Covance, 5, 7.5 or 10 mg/kg) twice weekly through intraperitoneal injection. When indicated, mice also received recombinant Aβ42 at 0.32 mg/kg twice weekly from day 10 post-implantation through intraperitoneal injection. Imaging was performed using an IVIS Spectrum imager (Xenogen). Animals were anesthetized with isoflurane and placed onto a warmed stage inside the camera box. Animals next received intraperitoneal luciferin (200 mg/kg) 5 min prior to imaging. For quantification, rectangular regions of interest incorporating the entire animal were measured. The signal was measured in photons per second using Living Image software. Mice were sacrificed when moribund. At autopsy, tumours were excised and histologically confirmed. Mice were examined for metastases, which were confirmed both through H&E staining and specific human nuclei staining with MAB1281 antibody (Sigma). Experiments were carried out under blinded conditions.

**Gene expression analyses**

The following datasets were retrieved from public sources: (i) for prostate cancer: E-MTAB-6128 (n=101) and TCGA; subtype classification was performed using the PAM50 classifier [6]; (ii) for colon cancer: GSE39582 (“CIT cohort”, n=566), GSE13294, GSE18088, GSE14333, GSE13067, GSE17536, GSE17537, GSE33113, GSE26682, TCGA, altogether forming the “validation cohort” (n=1647); subtype classification systems assignments were performed using original published predictor methods as described in [7].

**Statistical analysis**

The results from experimental data are reported as the means ± standard errors of the means (s.e.m.). Unpaired non parametric tests were used for group comparisons, as appropriate. Analyses involving two groups were carried out using the Mann-Whitney rank-sum test. Analyses involving >2 groups were carried out using the Kruskal-Wallis test followed by post-hoc Wilcoxon rank-sum tests with Holm’s correction for multiple comparisons. Results from RNA analysis in cohorts are expressed as median and interquartile range. Statistical analysis was performed in R using one-way ANOVA followed by Wilcoxon rank-sum tests with Holm’s correction for multiple comparisons. Survival curves for mice experiments were analysed with GraphPad Prism using the log-rank test. Survival curves were obtained using Kaplan-Meier estimates using the “survival” [(Cancer Genome Atlas Network, 2012)](http://cran-r-project.org/web/packages/survival/index.html) R package and differences between groups of patients were assessed using the log-rank test for univariate analyses or Cox models for multivariate analyses. A *p*-value < 0.05 was considered significant.

**Legends to Supplementary Figures**

**Supplementary Figure 1:** Pearson correlation coefficient between mRNA expression of *PRNP* and that of *CAV1* mRNA (**A**) or CAV1 protein (**B**) in various patient cohorts (grey bars) analysed through the Morpheus platform at the Broad Institute (<https://software.broadinstitute.org/morpheus/> )and cancer cell line panels (white bars) (PRAD: prostate adenocarcinoma, READ: rectal adenocarcinoma, BRCA: breast cancer, COAD: colon adenocarcinoma, PAAD: pancreatic adenocarcinoma, LUAD: lung adenocarcinoma, GBM: glioblastoma, OV: ovarian cancer, CCLE: cancer cell line encyclopaedia [8], NCI-60: National Cancer Institute panel [9]). **C** Pearson correlation coefficient between mRNA expression of *APP* and that of *PRNP* and *CAV1* mRNA and CAV1 protein in the CCLE.

**Supplementary Figure 2:** Protein analyses and their quantification. **A-B** Quantification of Western blot analysis of PrP^C^, CAV1 and APP protein expression in *PRNP*-silenced versus control PC3 prostate cancer cells displayed in Figures 1C and 2B. **C-D** Quantification of Western blot analysis of PrP^C^, CAV1 and APP protein expression in *PRNP*-silenced versus control MDST8 colon cancer cells in Figures 1D and 2D. Results are expressed as means of n=2 independent triplicates of cell preparations (except for **B** n=2 independent duplicates of cell preparations) ± s.e.m. * *p*<0.05, ** *p*<0.01 vs. control (Mann-Whitney test). **E** Western blot analysis of PrP^C^ expression in LoVo cells transfected with the pcDNA3-prnp plasmid that expresses mouse *Prnp* 3 days (left panel) or 5 days (right panel) post-transfection. Two representative replicates of each condition are shown. TAZ was used as loading control.

**Supplementary Figure 3:** Identification of *DKK1*, *DKK3* and *PDGFC* as genes whose expression is significantly correlated to that of *APP* mRNA, CAV1 mRNA and protein or *PRNP* mRNA in the CCLE.

**Supplementary Figure 4:** Impact of cell exposure to anti-prion 6D11 or Sha31 antibodies. **A** *BACE1*, *CAV1*, *IDO1*, *TGFB1* and *ZEB1* mRNA levels were measured in cell extracts of MDST8 colon cancer cells exposed to 6D11 antibodies versus control isotype antibodies. **B** qRT-PCR analysis of *DKK1* and *PDGFC* mRNA levels in PC3 prostate cancer cells exposed to Sha31 antibodies versus control isotype antibodies. **C** qRT-PCR analysis of *DKK3* and *PDGFC* mRNA levels in MDST8 colon cancer cells exposed to Sha31 antibodies versus control isotype antibodies. Results are expressed as means of n=2 independent replicates of cell preparations (n=6 for **A** and **C** and n=5 for **B**) ± s.e.m. * *p*<0.05, ** *p*<0.01 vs. control (Mann-Whitney test).

**Supplementary Figure 5:** Imaging of mice and metastases

**A-B**. Bioluminescence imaging of PC3 xenografts in Sham **(A)**- or 6D11 (10 mg per kg)- treated **(B)** mice at day 35. **C**. Metastases were histologically confirmed through hematoxylin and eosin (left) as well as human nuclei staining (right).

**Supplementary Figure 6:** Relative *BACE1*, *CAV1*, *DKK1*, *DKK3*, *PDGFC*, *PRNP*, *TGFB1* and *ZEB1* gene expression in prostate cancer patients of the E-MATB6128 dataset in Basal, Luminal A (LumA) and Luminal B (LumB) molecular subgroups according to the PAM50 classification by Zhao [6]. The Wilcoxon rank-sum test was used for group comparisons.

**Supplementary Figure 7:** Relative *BACE1*, *CAV1*, *DKK1*, *DKK3*, *PDGFC*, *PRNP*, *TGFB1* and *ZEB1* gene expression in prostate cancer patients of the TCGA dataset in Basal, Luminal A (LumA) and Luminal B (LumB) molecular subgroups according to the PAM50 classification by Zhao [6]. The Wilcoxon rank-sum test was used for group comparisons.

**Supplementary Figure 8:** Relative *BACE1*, *CAV1*, *DKK3*, *PDGFC*, *PRNP*, *TGFB1* and *ZEB1* gene expression in colon cancer patients of the GSE39582 dataset according to the CMS classification by Guinney [7]. The Wilcoxon rank-sum test was used for group comparisons.

**Supplementary Figure 9:** Kaplan-Meier overall survival (OS) (left panel) and relapse free survival (RFS) (right panel) according to high and low *BACE1* gene expression was determined in colorectal cancer patients of the validation cohort. Hazard ratios were adjusted for sex, age at diagnosis and TNM stage.

**Supplementary Table 1**

| gene | Forward primer | Reverse primer |
| --- | --- | --- |
| APP | TCTCCCTGCTCTACAACGTG | TTTCCGTAACTGATCCTTGG |
| BACE1 | ACCACCAACCTTCGTTTGCCC | AAGGGGTGGTGCCTGCTTGC |
| CAV1 | AGCCGTGTCTATTCCATCTA | TTTCTTTCTGCAAGTTGATG |
| DKK1 | CGCCGAAAACGCTGCAT | TTTCCTCAATTTCTCCTCGGAA |
| DKK3 | GCTTCTGGACCTCATCACCTG | TCGGCTTGCACACATACACC |
| IDO1 | GATGTCCGTAAGGTCTTGCC | TGCAGTCTCCATCACGAAAT |
| PDGFC | TGTCATGCCACAATTCACAG | TGCCATCTCTCTGGTTCAAG |
| PRNP | CGAGCTTCTCCTCTCCTCAC | GTTCCATCCTCCAGGCTTC |
| RPL13A | CCTGGAGGAGAAGAGGAAAGAGA | GAGGACCTCTGTGTATTTGTCAA |
| TGFB1 | ATGGTGGAAACCCACAACG | GCTGAGGTATCGCCAGGAAT |
| ZEB1 | AAGAATTCACAGTGGAGAGAAGCCA | CGTTTCTTGCAGTTTGGGCATT |

**Supplementary references**

1. Bateman RJ, Munsell LY, Chen X, Holtzman DM, Yarasheski KE. Stable isotope labeling tandem mass spectrometry (SILT) to quantify protein production and clearance rates. J Am Soc Mass Spectrom. 2007;18:997–1006.

2. Le Corre D, Ghazi A, Balogoun R, Pilati C, Aparicio T, Martin-Lannerée S, et al. The cellular prion protein controls the mesenchymal-like molecular subtype and predicts disease outcome in colorectal cancer. EBioMedicine. 2019;46:94–104.

3. Bray NL, Pimentel H, Melsted P, Pachter L. Near-optimal probabilistic RNA-seq quantification. Nat Biotechnol. 2016;34:525–7.

4. Pimentel H, Bray NL, Puente S, Melsted P, Pachter L. Differential analysis of RNA-seq incorporating quantification uncertainty. Nat Methods. 2017;14:687–90.

5. Subramanian A, Tamayo P, Mootha VK, Mukherjee S, Ebert BL, Gillette MA, et al. Gene set enrichment analysis: a knowledge-based approach for interpreting genome-wide expression profiles. Proc Natl Acad Sci U S A. 2005;102:15545–50.

6. Zhao SG, Chang SL, Erho N, Yu M, Lehrer J, Alshalalfa M, et al. Associations of Luminal and Basal Subtyping of Prostate Cancer With Prognosis and Response to Androgen Deprivation Therapy. JAMA Oncol. 2017;3:1663–72.

7. Guinney J, Dienstmann R, Wang X, de Reyniès A, Schlicker A, Soneson C, et al. The consensus molecular subtypes of colorectal cancer. Nat Med. 2015;21:1350–6.

8. Barretina J, Caponigro G, Stransky N, Venkatesan K, Margolin AA, Kim S, et al. The Cancer Cell Line Encyclopedia enables predictive modelling of anticancer drug sensitivity. Nature. 2012;483:603–7.

9. Reinhold WC, Sunshine M, Liu H, Varma S, Kohn KW, Morris J, et al. CellMiner: a web-based suite of genomic and pharmacologic tools to explore transcript and drug patterns in the NCI-60 cell line set. Cancer Res. 2012;72:3499–511.
