# Supplementary figures and images for: A proof of concept for targeting the PrPC - Amyloid β peptide interaction in basal prostate cancer and mesenchymal colon cancer
Source: Oncogene. 2022 Aug 12;41(38):4397–404. doi: 10.1038/s41388-022-02430-7 (PMC9481457; doi:10.1038/s41388-022-02430-7)

# Supplementary Figure 1

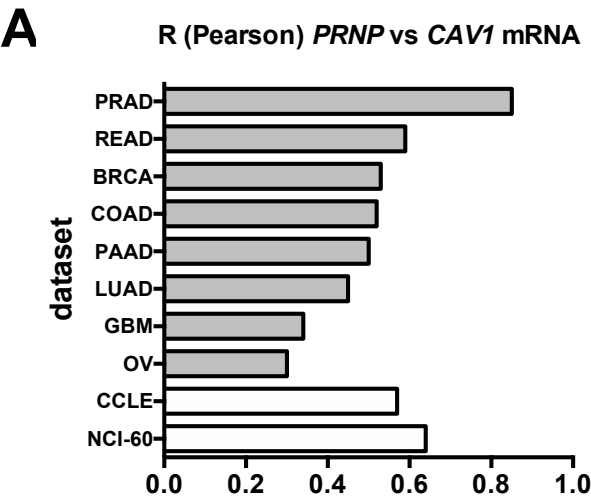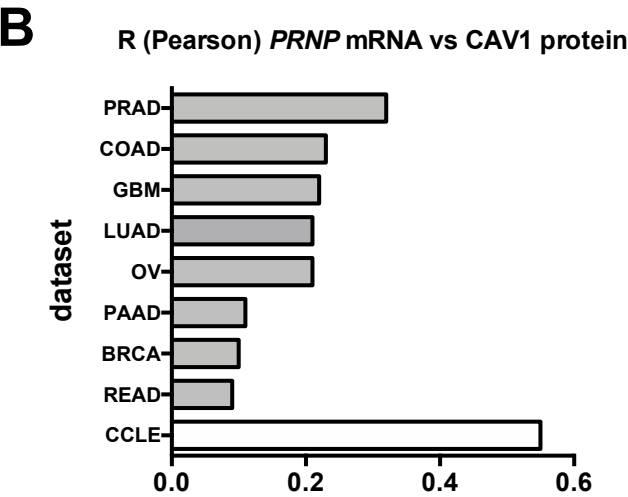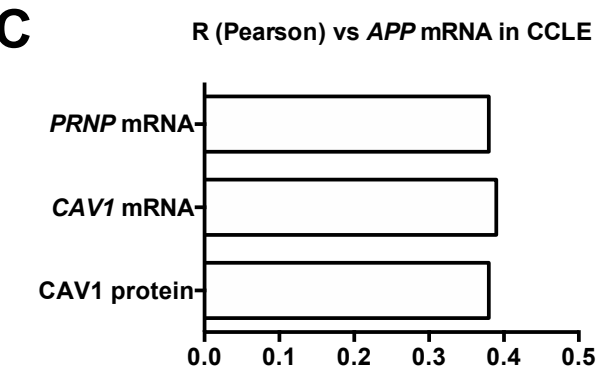

Supplement: Supplementary file 2 — supplementary Figure 1 [file 41388_2022_2430_MOESM2_ESM.pdf]

Supplementary Figure S2

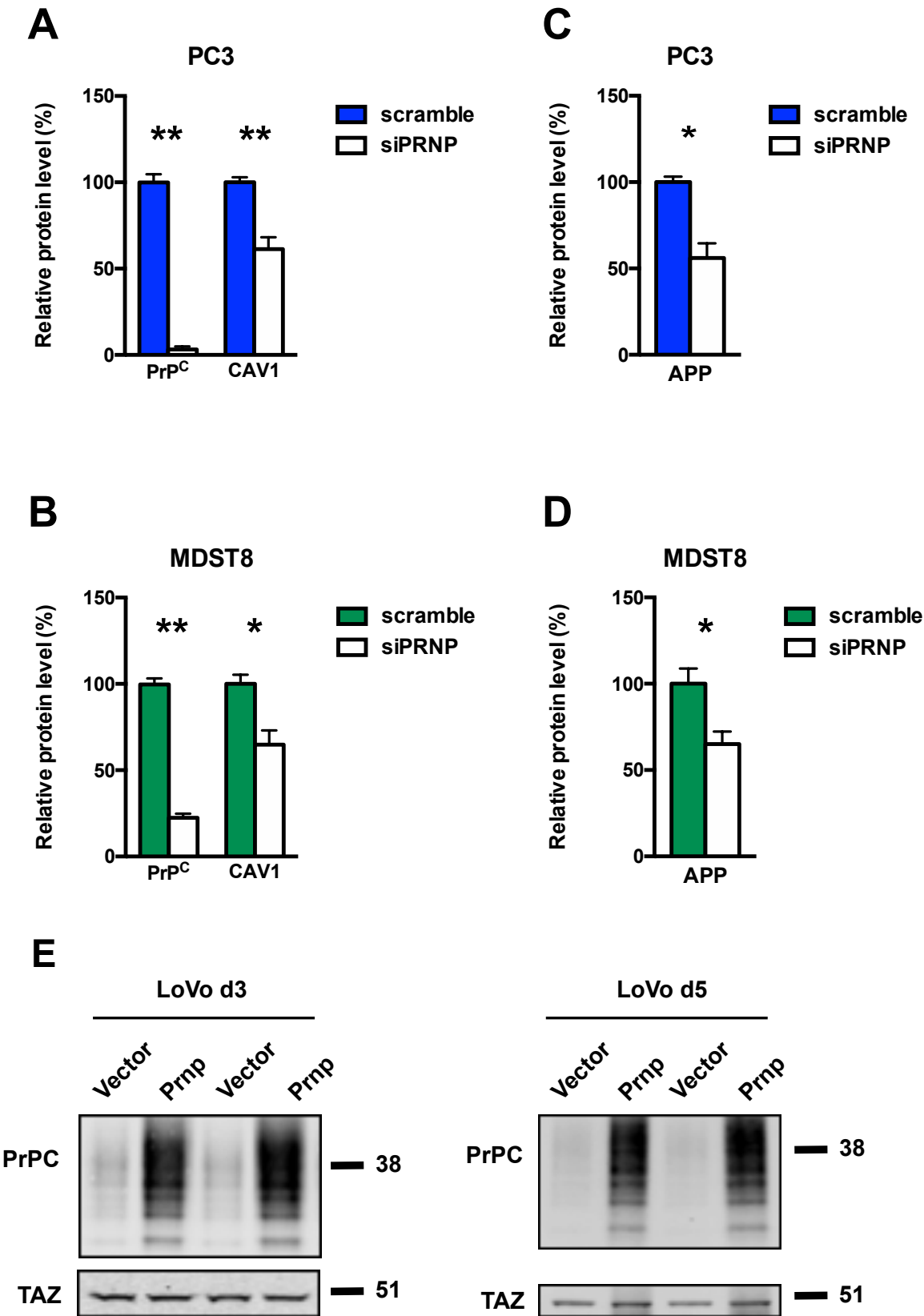

Supplement: Supplementary file 3 — supplementary Figure 2 [file 41388_2022_2430_MOESM3_ESM.pdf]

# Supplementary Figure 4

A

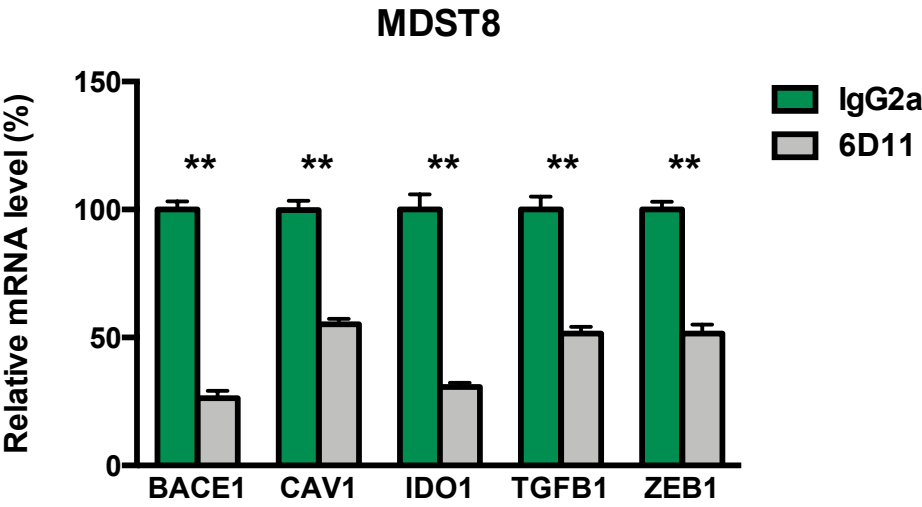

B

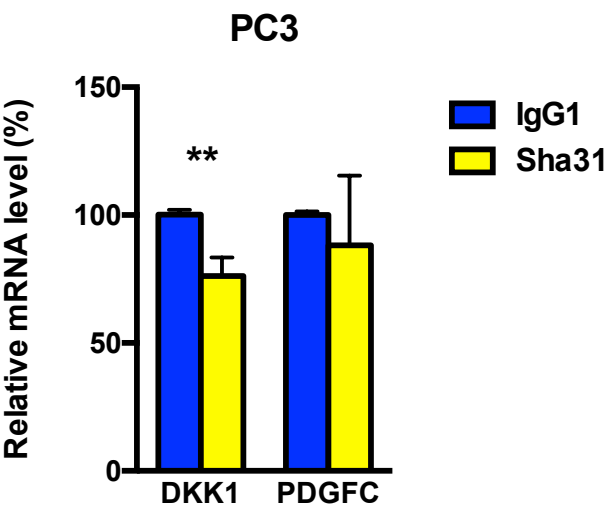

C

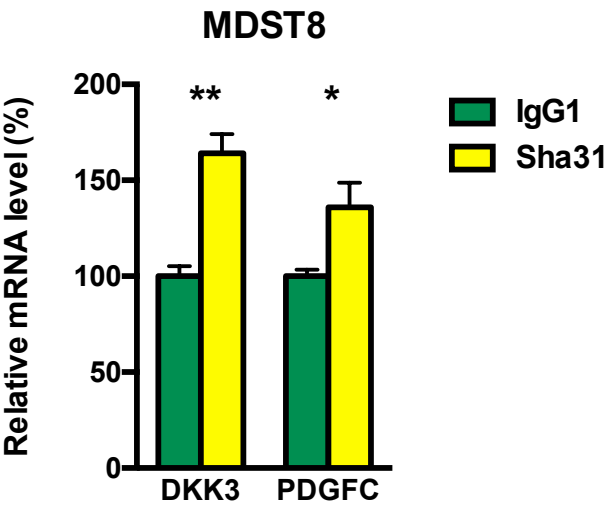

Supplement: Supplementary file 5 — supplementary Figure 4 [file 41388_2022_2430_MOESM5_ESM.pdf]

Supplementary Figure S5

A

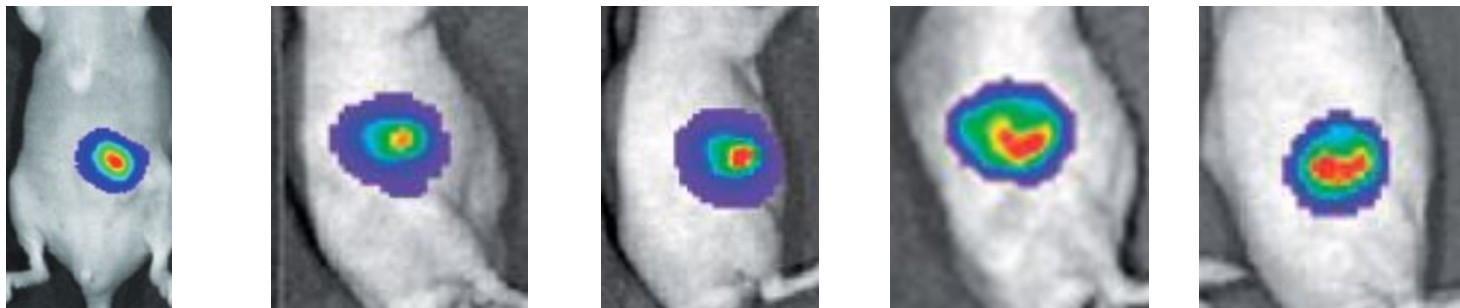

B

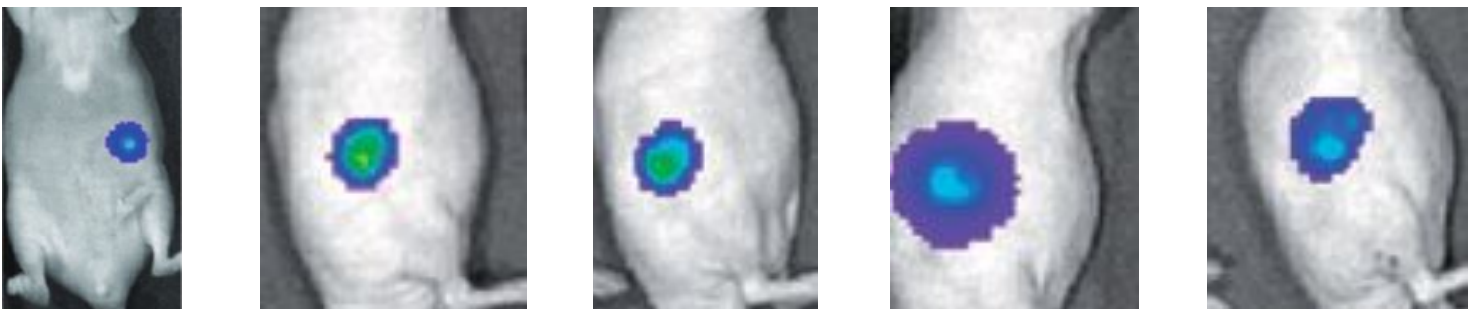

C

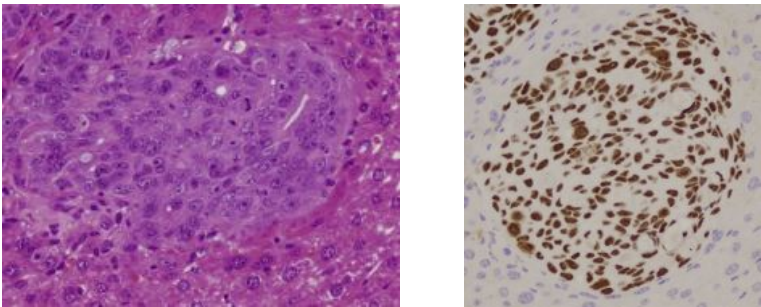

Supplement: Supplementary file 6 — supplementary Figure 5 [file 41388_2022_2430_MOESM6_ESM.pdf]

# Supplementary Figure S6

## Prostate cancer (E-MATB6128)

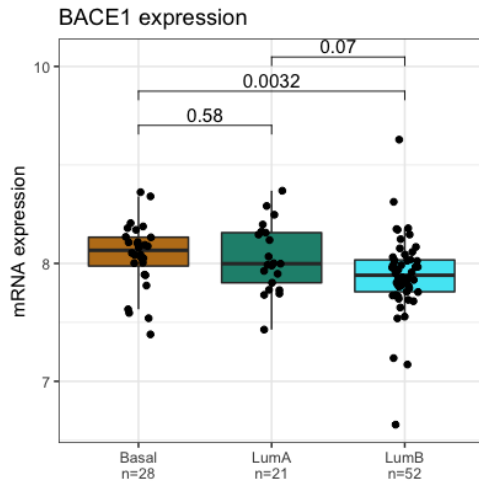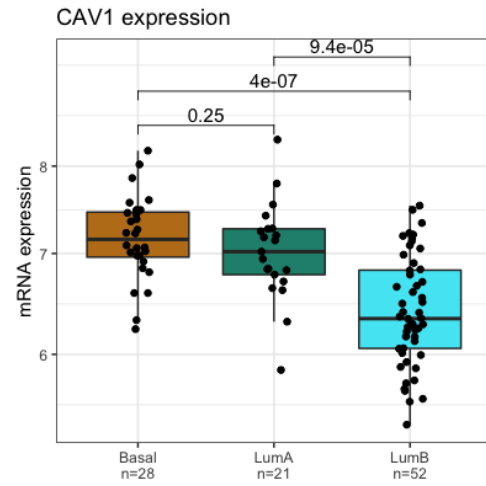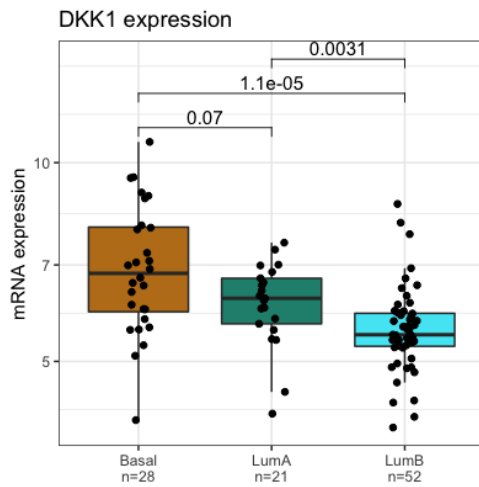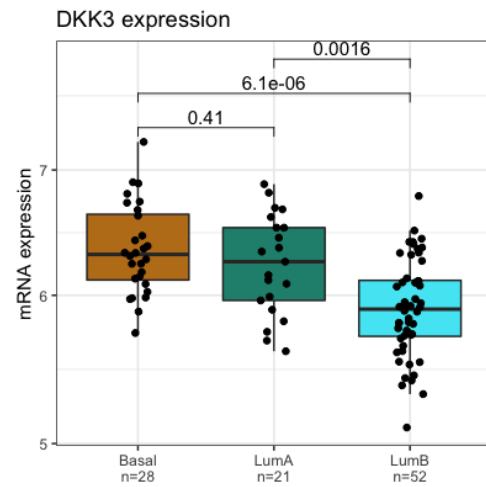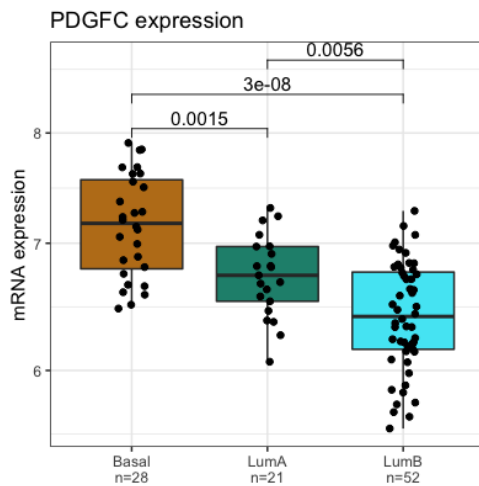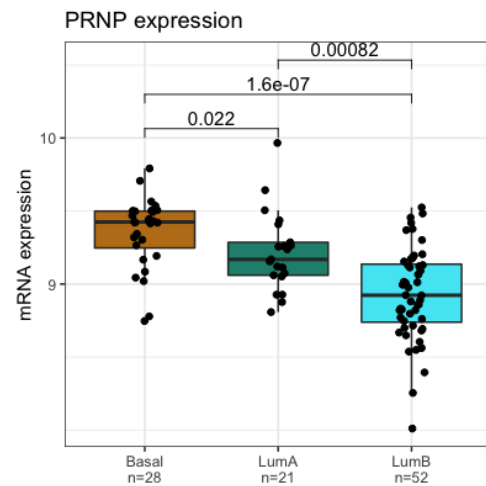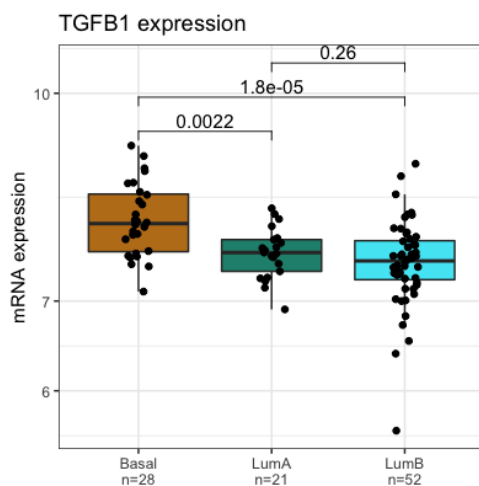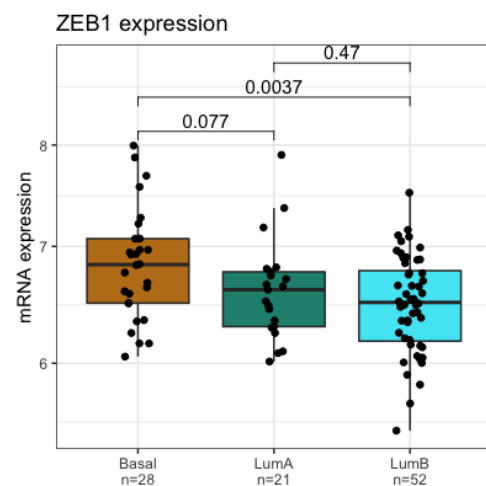

Supplement: Supplementary file 7 — supplementary Figure 6 [file 41388_2022_2430_MOESM7_ESM.pdf]

# Supplementary Figure S7

## Prostate cancer (TCGA)

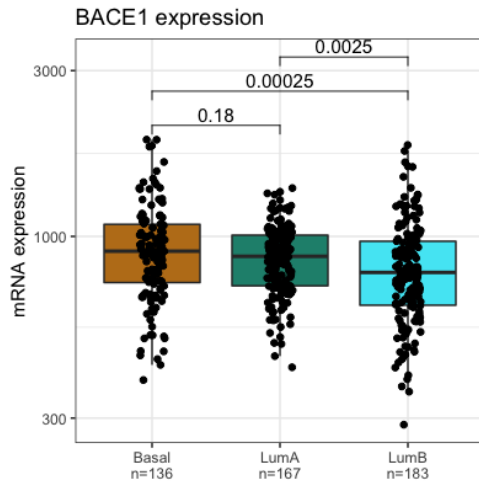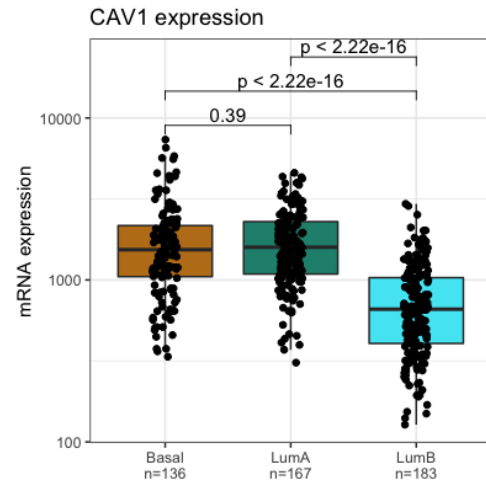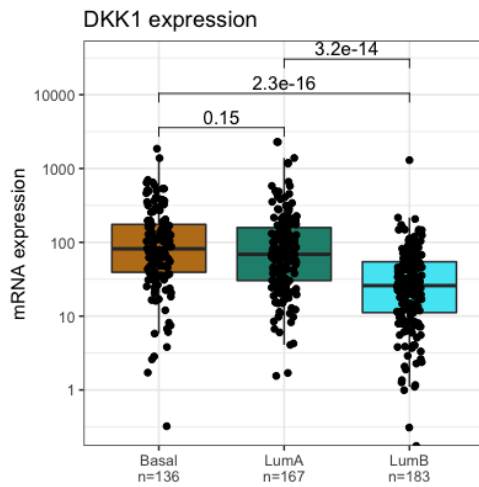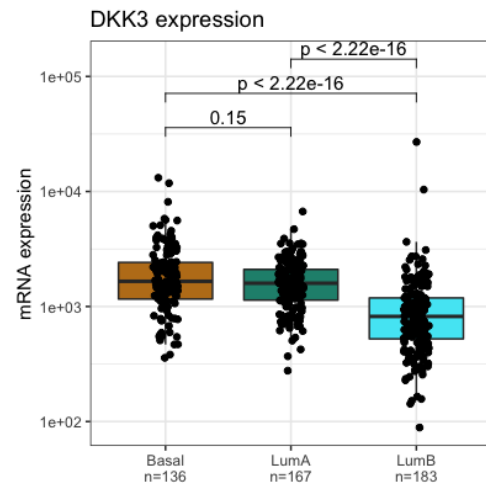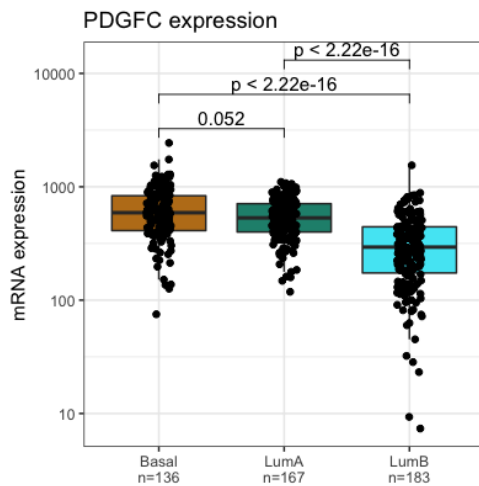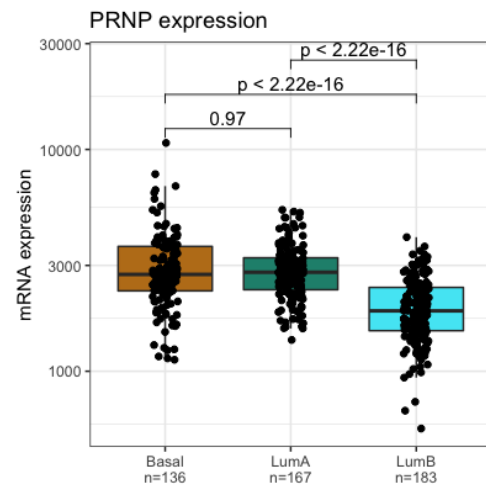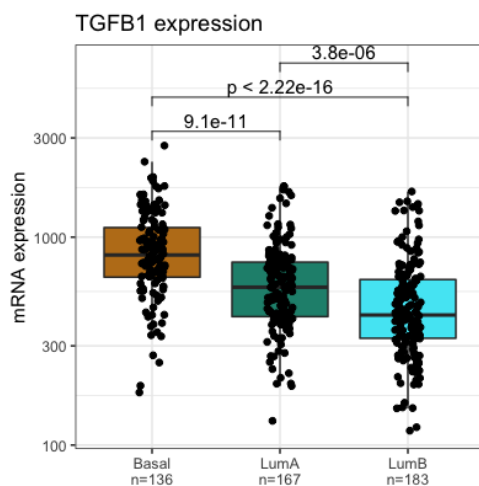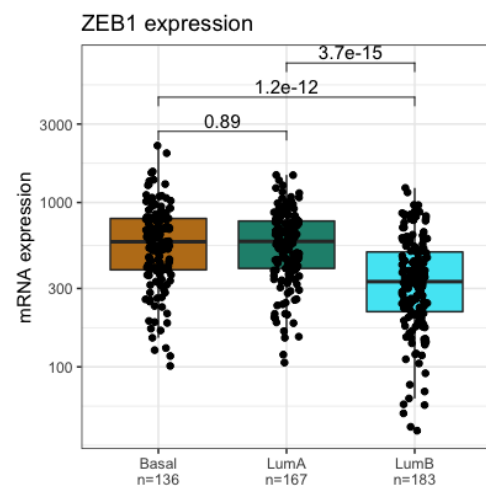

Supplement: Supplementary file 8 — supplementary Figure 7 [file 41388_2022_2430_MOESM8_ESM.pdf]

# Supplementary Figure S8

## Colorectal cancer (GSE39582)

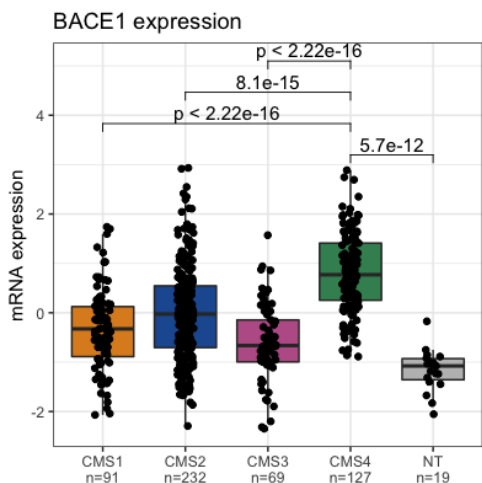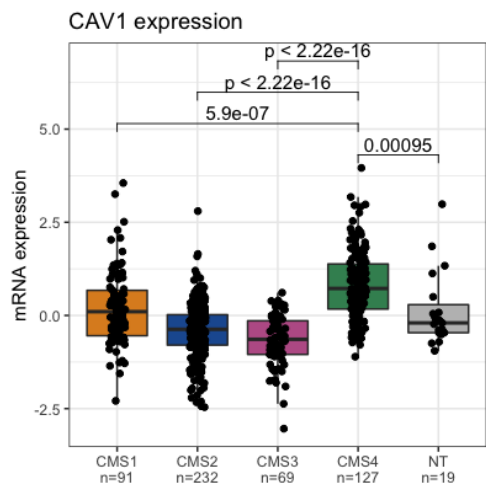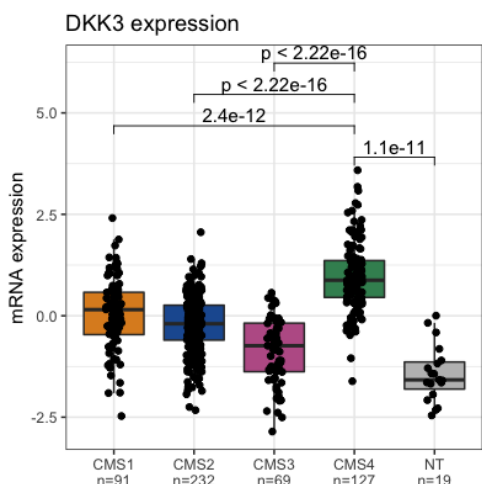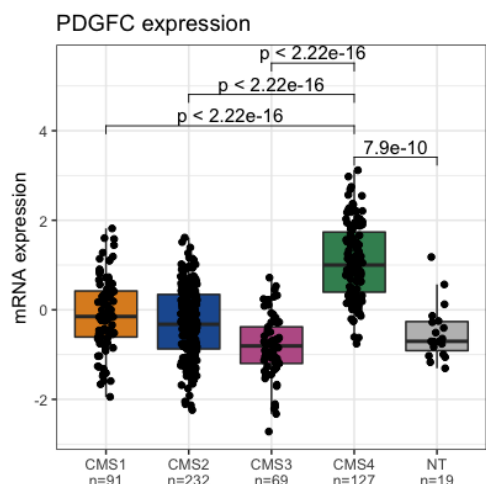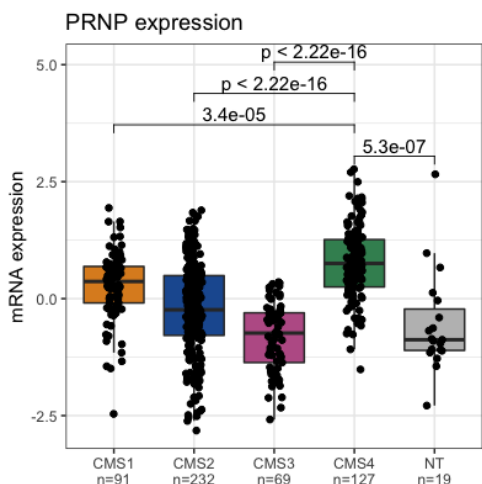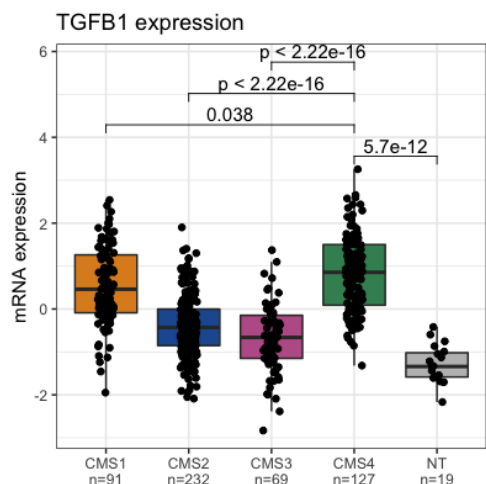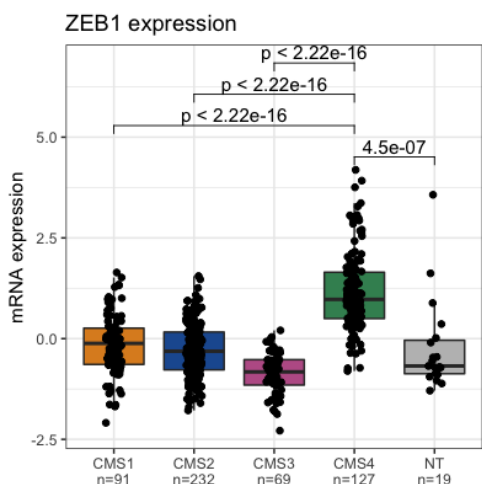

Supplement: Supplementary file 9 — supplementary Figure 8 [file 41388_2022_2430_MOESM9_ESM.pdf]

# Supplementary Figure S9

A

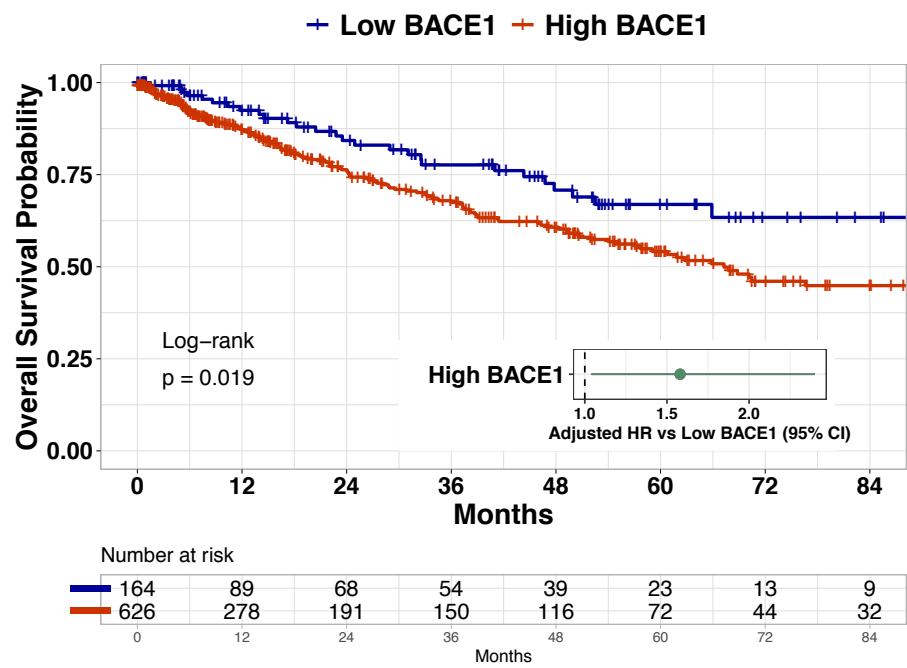

B

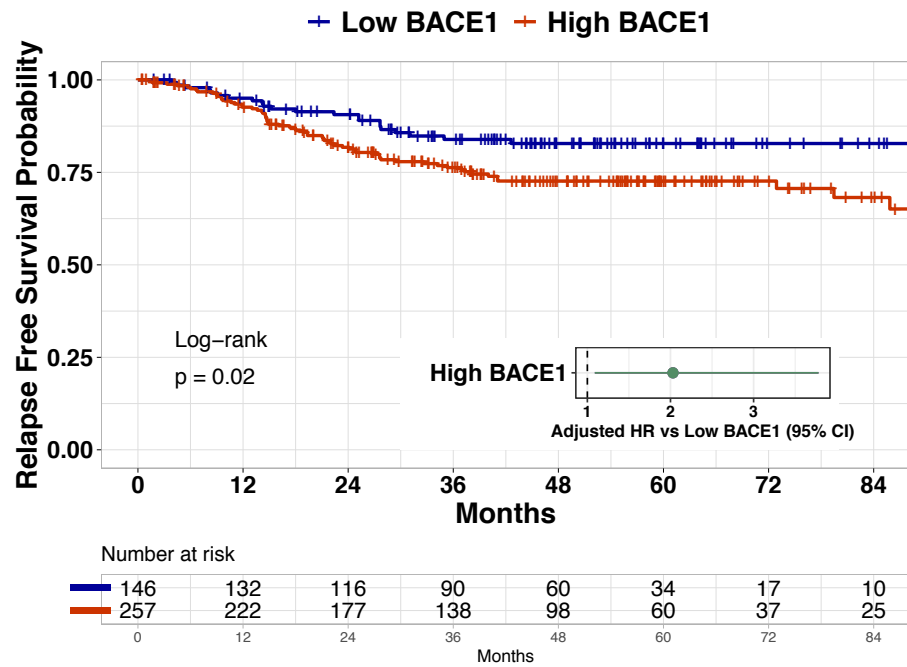

Supplement: Supplementary file 10 — supplementary Figure 9 [file 41388_2022_2430_MOESM10_ESM.pdf]
