# Supplementary material for: A proof of concept for targeting the PrPC - Amyloid β peptide interaction in basal prostate cancer and mesenchymal colon cancer
Source: Oncogene. 2022 Aug 12;41(38):4397–404. doi: 10.1038/s41388-022-02430-7 (PMC9481457; doi:10.1038/s41388-022-02430-7)

Supplementary Figure 3

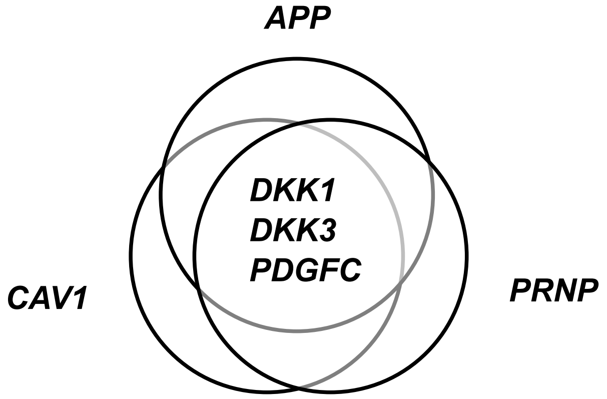

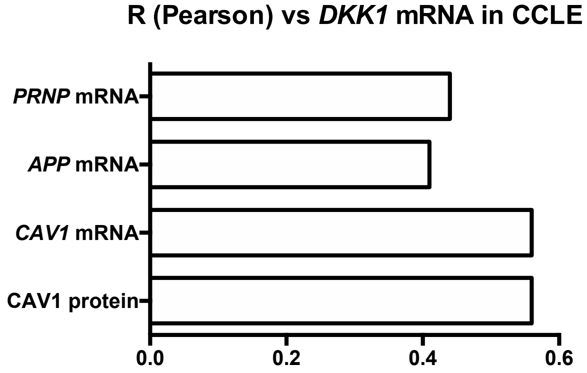

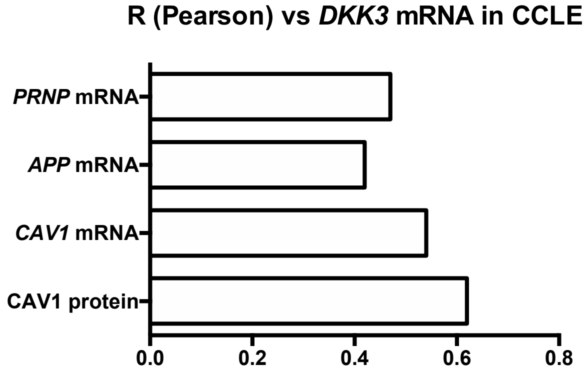

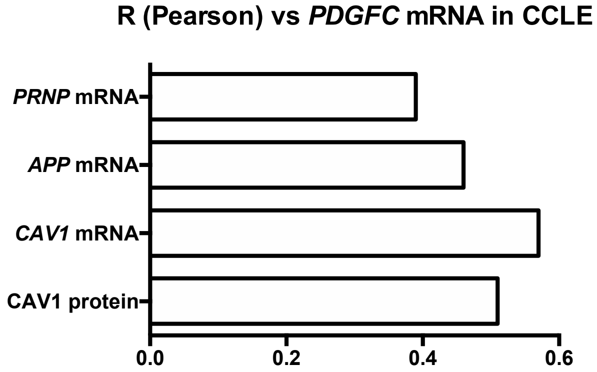

Supplement: Supplementary file 4 — supplementary Figure 3 [file 41388_2022_2430_MOESM4_ESM.pdf]
